# The roles and therapeutic implications of messenger RNA internal *N* ^7^‐methylguanosine and *N* ^6^‐methyladenosine modifications in chemoresistance

**DOI:** 10.1002/ctm2.1400

**Published:** 2023-09-04

**Authors:** Zhicong Zhao, Ying Qing, Xiaolan Deng, Rui Su, Jianjun Chen

**Affiliations:** ^1^ Department of Systems Biology Beckman Research Institute of City of Hope Monrovia California USA; ^2^ Department of Liver Surgery Renji Hospital School of Medicine Shanghai Jiao Tong University Shanghai China; ^3^ City of Hope Comprehensive Cancer Center City of Hope Duarte California USA

1

Resistance to chemotherapy is responsible for the death of most cancer patients. RNA modifications are key players in post‐transcriptional gene regulation and their dysregulations contribute to tumor initiation, progression, and resistance to chemotherapy. Despite the identification of over 170 chemical modifications in nearly all types of RNAs, the biological functions and underlying mechanisms of the vast majority of RNA modifications remain elusive in the context of tumorigenesis and drug resistance. *N* ^6^‐methyladenosine (m^6^A) is the most prevalent internal modification in eukaryotic messenger RNAs (mRNAs). The characterization of proteins that deposit, remove, and recognize mRNA m^6^A marks, as writers, erasers and readers, respectively, has revealed the profound roles of m^6^A in determining mRNA fates during both physiological and pathological processes. Besides m^6^A, another commonly observed positively charged modification is *N* ^7^‐methylguanosine (m^7^G), which is ubiquitously located at the 5′ cap of mRNAs. This m^7^G cap is fundamental to mRNA stability, export, and translation. Meanwhile, multiple independent studies have revealed that m^7^G modification could also be introduced internally onto mRNAs by the METTL1/WDR4 methyltransferase complex.^1^, ^2^ In eukaryotic cells, the internal m^7^G/G ratio in mRNAs ranges from 0.02% to 0.05%, roughly at 5%−10% of the level of mRNA m^6^A/A ratio. However, unlike m^6^A, the functions of mRNA internal m^7^G remain largely unexplored. As the role of internal m^7^G is mediated by its reader proteins, identification of such readers is pivotal for us to understand the function of internal m^7^G. Our recent work discovered the Quaking (QKI) protein (including three isoforms, QKI5, QKI6 and QKI7) as the first reader for internal m^7^G modification.^3^ Utilizing multiple high‐throughput sequencing approaches, we demonstrated that QKI7 regulates the stability and translation efficiency of a subset of internal m^7^G‐modified transcripts under stress conditions, rendering cancer cells more responsive to chemotherapy drugs in vitro and in vivo.

Tumours are complex ecosystems that dynamically adapt to various types of external stress stimuli. Evidence is emerging that RNA modifications serve as critical regulatory mechanisms in cancer cells to respond to external stress, including chemotherapeutics, and are critical for tumorigenesis and drug resistance. Consistently, the expression of their regulators (i.e., writers, erasers, and readers) is frequently dysregulated in tumours. Notably, many genes involved in drug resistance are decorated with m^6^A on their transcripts, including drug‐metabolizing enzymes (e.g. CYP2C8), multidrug efflux transporters (e.g., ABCG2, ABCC9 and ABCC10), and DNA damage repair genes (e.g. p53, BRCA1).^4^ Additionally, a recent transcriptome‐wide profiling study of internal m^7^G revealed a significantly lower internal m^7^G level on ABC transporter‐encoded transcripts (key players in multidrug resistance) in drug‐resistant acute myeloid leukaemia (AML) cells than in regular AML cells.^5^ Therefore, targeting the dysregulated m^6^A/m^7^G machinery appears to be a promising strategy to overcome cancer chemoresistance (Figure 1).

**FIGURE 1:**
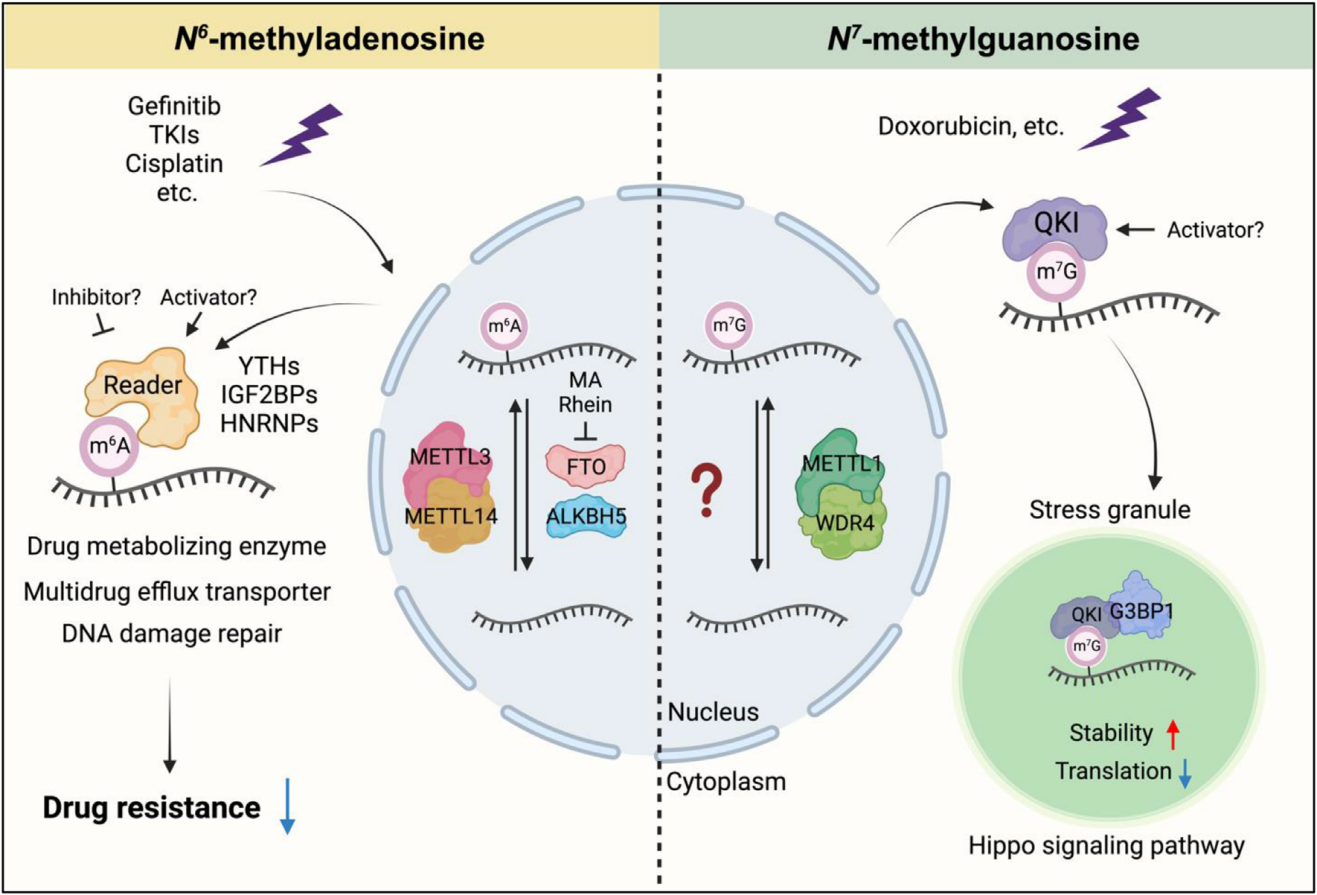
Targeting *N* ^6^‐methyladenosine (m^6^A) and *N* ^7^‐methylguanosine (m^7^G) regulators to overcome drug resistance. Several small molecule drugs targeting m^6^A regulators (e.g. METTL3, METTL14, FTO and ALKBH5) have the potential to overcome drug resistance by regulating the expression of drug‐metabolizing enzymes, multidrug efflux transporters and drug‐mediated DNA damage repair transcripts. Additionally, Quakings (QKIs) play important roles as mRNA internal m^7^G‐binding proteins in modulating cancer cells’ response to chemotherapy drugs (e.g. doxorubicin). Thus, small molecule drugs targeting m^7^G regulators (e.g. METTL1, WDR4 and QKI) may also be a promising strategy to overcome drug resistance. This figure was created with BioRender.com.

Stress conditions including chemotherapy drugs induce the formation of stress granules (SGs), the membraneless cytoplasmic ribonucleoprotein particles in cells. The formation of SGs requires certain RNA binding proteins such as G3BP1 to act as a molecular hub to trigger SG assembly.^6^ Current evidence supports the implication of SGs in chemoresistance. For instance, *G3BP1* depletion sensitizes glioblastoma cell lines to Bortezomib treatment by suppressing SG assembly and glutamine deprivation overcomes chemoresistance in pancreatic cancer through inhibition of SG formation. However, the capacity of SG to confer cancer cells chemoresistance and survival advantage relies on the recruitment of a set of target RNAs to influence their expression/function. Until recently, the mechanism behind such recruitment has been mostly unknown. In 2020, Fu et al. reported that YTHDF proteins, as m^6^A readers, recruit m^6^A‐modified mRNAs into SGs by promoting phase separation.^7^ Our recent work revealed that, via direct interaction with the SG core protein G3BP1, QKI7 could selectively shuttle internal m^7^G‐modified mRNAs into SGs under stress.^3^ Importantly, the clinical relevance of this finding was demonstrated in cancer cells treated with doxorubicin, a first‐line chemotherapy drug that induces SG assembly. Further in vitro and in vivo models showed that forced expression of QKI7 sensitizes cancer cells to doxorubicin treatment, as associated with suppressed translation of a subset of m^7^G‐modified transcripts, including those in the Hippo signaling pathway (e.g. *GSK3B* and *TEAD1*) (Figure 1). Furthermore, clinical data from TCGA database showed a significant positive correlation between QKIs and several SG markers in various types of cancers, suggesting a broad role of QKIs as mRNA internal m^7^G‐binding proteins in regulating cancer cells’ stress response and drug resistance.

Given the critical roles of RNA modifications in cancer chemoresistance, researchers have enthusiastically explored the potential of targeting these RNA modification modulators to enhance the efficacy of chemotherapeutics. Some encouraging results have been achieved for small molecules targeting m^6^A regulators in different cancer types. For example, meclofenamic acid, an FTO inhibitor, restores gefitinib sensitivity in non‐small cell lung cancer, and the combination of rhein (another FTO inhibitor) with tyrosine kinase inhibitors (TKIs) may benefit tyrosine kinase inhibitor‐resistant cancer patients.^8^ In terms of m^7^G regulators, while our work highlights the significance of QKIs as internal m^7^G readers in drug resistance, several additional studies underscore the importance of m^7^G writers METTL1/WDR4 in chemoresistance.^9^ However, no small molecules targeting QKIs or METTL1/WDR4 have been reported so far. Thus, the development of potent therapeutic agents targeting m^7^G regulators represents a major area of research and potential advancement in cancer treatment.

While targeting RNA modifications holds promise as a novel therapeutic strategy to combat chemoresistance, our understanding of the fundamental molecular mechanisms underlying the functions of epitranscriptomic modifications, especially those other than m^6^A, in drug resistance is still in its infancy. Future efforts to fully elucidate the mechanism through which mRNA modifications (e.g. m^7^G and m^6^A) regulate the response of cancer cells to chemotherapy drugs, along with the development of novel potent therapeutic agents guided by these mechanisms and validated through clinical trials, are imperative. These endeavours may offer valuable insights into effectively overcoming drug resistance in clinical settings.

## CONFLICT OF INTEREST STATEMENT

The authors declare no conflict of interest.
